# The Effect of Single and Triple Testicular Biopsy Using Biopty Gun on Spermatogenesis in Pubertal Rats

**DOI:** 10.3390/ani11061569

**Published:** 2021-05-27

**Authors:** Tomislav Šušnjar, Ivana Kuzmić Prusac, Ivan Švagelj, Anđela Jurišić, Tomislav Šušnjar, Antonija Jurišić, Miro Jukić, Zenon Pogorelić

**Affiliations:** 1 Department of Pediatric Surgery, University Hospital of Split, 21000 Split, Croatia; tomislav.susnjar@optinet.hr (T.Š.); mirojukic.mefst@gmail.com (M.J.); 2 Department of Pathology, Forensic Medicine and Cytology, University Hospital of Split, 21000 Split, Croatia; ivanakp@mefst.hr; 3 Department of Pathology and Cytology, General County Hospital Vinkovci, 32100 Vinkovci, Croatia; svagelj.ivan@gmail.com; 4 Department of Cardiology, University Hospital Dubrava, 10000 Zagreb, Croatia; andjelajurisic1@gmail.com; 5 Department of Otorhinolaryngology and Head and Neck Surgery, University Hospital Dubrava, 10000 Zagreb, Croatia; tomosusnjar@gmail.com; 6 Dental Clinic Jurišić, 88240 Posušje, Bosnia and Herzegovina; antonija.jurisic1@gmail.com; 7 Department of Surgery, School of Medicine, University of Split, 21000 Split, Croatia

**Keywords:** testis, biopty gun, testicular biopsy, rat, semen analysis, needle biopsy

## Abstract

**Simple Summary:**

Nowadays, a punch biopsy is a simple, reliable and inexpensive method for different types of tissue sampling. Equally, it is the method of choice for obtaining testicular tissue samples for pathohistological analysis and sperm for intracytoplasmic sperm injection. The results of this study clearly showed that a single biopsy has little effect on the biopsied testis, especially on total fertility. Triple biopsy showed by the same parameters that histological and immunohistochemical consequences were more significant compared to single but without a significant effect on overall fertility. Sperm analysis showed that single and triple biopsies did not have a significant effect on sperm count, motility and morphology. In addition, both single and triple punch biopsies of one testicle did not significantly affect the overall fertility potential of pubertal rats.

**Abstract:**

Background: The aim of this study was to compare consequences in single and triple testicular biopsy by biopty gun in pubertal rats using histological and immunohistochemical analysis. Methods: Thirty-two Sprague-Dawley male rats were used as the experimental model. The rats were randomly divided into three study groups. The rats from the first group (*n* = 12) received a single-biopsy of upper pole of the left testis, while the rats from the second group (*n* = 10) received triple-biopsy of upper and lower poles and lateral surface of left testis. The third group (*n* = 10) was a control group. On the eightieth day after the biopsy in all rats bilateral orchiectomy and funiculectomy were performed to obtain testicular tissue and sperm for analysis. The consequences of the puncture were observed by pathohistology, immunohistochemistry and semen analysis. Results: The results of the study showed lower percentage of sperm count (14.5 mill/mL vs. 16 mill/mL, *p* = 0.130), sperm motility (24.6% vs. 32.7%, *p* > 0.05), abnormal sperm (30% vs. 27%, *p* > 0.05), atrophic tubules (21% vs. 6%, *p* < 0.001), volume (1.7 mL vs. 2.28 mL, *p* < 0.01) and apoptotic index (1.56 vs. 1.19, *p* = 0.650) in the testes with a triple-biopsy compared to the testes with a single-biopsy. Semen analysis showed a borderline significant difference between the group with triple-biopsy where sperm count was lower than it in the control group (14.5 mill/mL vs. 17.5 mill/mL, *p* = 0.05). A single-biopsy has little effect on the testis, especially on overall fertility. A triple-biopsy showed higher degree of the testicular damage but without a significant impact on overall fertility. Semen analysis showed that single- and triple-biopsies did not have a significant effect on sperm count, motility and morphology. Conclusion: Biopty gun procedure is a cheap, simple and reliable method for testicular biopsy in rats without a significant effect on sperm count, motility and morphology.

## 1. Introduction

Testicular biopsy is an important diagnostic procedure and considered one of key procedures in male infertility workup. It is most commonly used in distinction of obstructive azoospermia and non-obstructive azoospermia and for sperm extraction for intracytoplasmic sperm injection (ICSI). In addition, it is a very important diagnostic tool often used in case of suspected tumors [1]. Male infertility can occur as a consequence of different pathological conditions such as cryptorchidism, varicocele, testicular torsion, mumps, etc. In these cases, correct diagnosis is of crucial significance because proper spermiogram from ejaculate can be acquired only 29–33 months after the beginning of puberty [2]. Objective insight of the morphological condition of testicular tissue can be defined by testicular biopsy. Testicular biopsy in children, with all its controversies, can be very useful in patients with cryptorchidism or varicocele and in diagnosing carcinomas in situ (CIS) [3,4,5,6]. The only way to accurately prove the success of hormonal therapy with gonadotropin-releasing hormone and human chorionic gonadotropin or to evaluate testicular status after which supplemental hormonal therapy may be started is the testicular biopsy which may be performed during the orchidopexy [3,4,5,6]. It is well known that varicocele can cause certain histological changes on the testis. Benefit of biopsy is to determinate the patients who will have a positive effects from the surgery. While the routine indication of a diagnostic testicular biopsy before varicocele repair in nonobstructive azoospermic (NOA) men is controversial and not without its own risks, it plays a realistic and important role as most men with NOA will still require IVF with intracytoplasmic sperm injection (ICSI) to pursue a biological pregnancy [1,2,3,4,5,6]. Testicular biopsy is also useful for defining damage in testicular torsion or other traumas of testicular tissue as well as for the follow-up in condition during the chemotherapy regime in acute lymphatic leukemia [3,7].

Open testicular biopsy was first introduced by Charny in 1940 [8,9]. In 1984 Cohen introduced needle biopsy, while Rajfer and Binder first introduced biopty gun in 1989 [8,10]. Testicular biopsy with biopty gun was shown as safe and fast method, with acquiring adequate testicular tissue for the analysis in correlation with open biopsy. Percutaneous testicular needle biopsy is a quick, simple and safe diagnostic procedure. Speed in which needle passes through testicular tissue does not create significant pain or discomfort [8,10,11,12,13,14]. At the end of the 1990s, open biopsy was used more frequently. The harmfulness of this method has been showed through early complications, such as inflammation, extraction site hematoma, which diminished six months after leaving linear scar tissue and calcifications behind [15]. Later, many studies have shown benefits of needle, compared to open biopsy. Nowadays, a biopty gun is often used for a number of indications. Most frequently it has been used in evaluation of infertility (to distinguish non-obstructive azoospermia from obstructive) and taking sperm for ICSI. It is also important in children with cryptrochism, testicular injury and torsion, varicocele as well as for the diagnosis of malignant diseases. Regarding histological changes as a consequence of open and needle biopsy it was shown that sixty days after the biopsy inflammatory changes vanquished, there was no scar formations and no tubular hyalinisation, but there were calcifications as a dominant histological finding in both groups [16]. Pathological changes in single biopsy were significantly lower than in triple biopsy using the testicular sperm extraction (TESE) and testicular fine-needle aspiration (TEFNA) and there were no changes in contralateral testicular tissue using histological analysis [17,18]. Open biopsy in prepubertal time has no significant influence on later spermogenesis and fertility in rats [3].

The aim of this study was to compare consequences in single and triple testicular biopsy by biopty gun spring-loaded needle in pubertal rats using histological and immunohistochemical analysis.

## 2. Materials and Methods

### 2.1. Experimental Animals

Thirty-two Sprague-Dawley male rats were used as the experimental model for this study. The rats were housed in the barn of the University of Split, School of medicine, two in a cage at a temperature of 20 ± 5 °C and a humidity of 55 ± 5%. The day/night cycle was set at 10/14 h. There were no food/water restrictions. The study protocol was approved by the Ethics Review Board of University of Split, School of Medicine (reference 2181-198-03-04/10-12-0002) and Ministry of agriculture (reference 525-10/0255-12-2). At around the age of forty days, rats (weighing about 150 g) were randomly divided into three experimental groups. The first group (single-biopsy) was consisted of twelve rats while the second (triple-biopsy) and third (control) groups were consisted of ten rats, each. In compliance with the study of Nakane et al. [3], increase of apoptotic index was expected in both experimental groups after single and triple-biopsy. To prove a statistically significant change of apoptotic index in the biopsied groups compared with the control group at the significance level a z = 0.05 and for the test strength of 80%, 10 rats were required per group. 

### 2.2. Surgery

**Figure 1:**
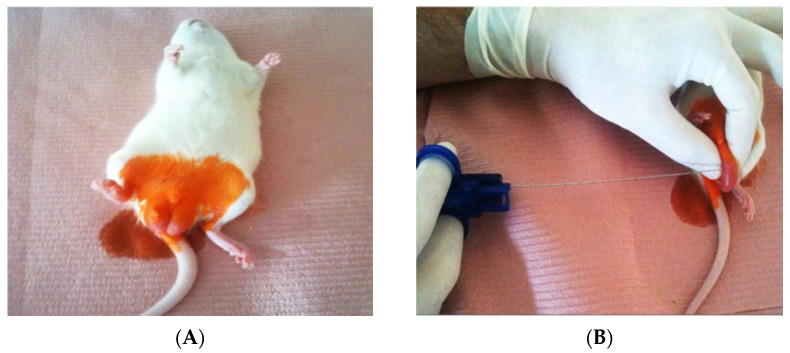
Intraoperative finding during the punch biopsy: (**A**)—Anesthetized rat after preparation of the operating field; (**B**)—Puncture of the testes using biopty gun.

Animals from the first two groups were anesthetized by intraperitoneal urethane injection (20% solution, 1 mL/100 g body weight). The rats from the first group underwent single punch biopsy of the upper pole of the left testis, while the rats from the second group underwent a triple punch biopsy of the left testis. The biopsies were performed at the upper and lower poles and lateral surface of left testis. The biopsies were taken under the aseptic conditions. Skin disinfection was performed using a stained antiseptic (SkinDes^®^, Antiseptica GmbH, Pulheim, Germany) (Figure 1A). A biopty gun (Spring Loaded Biopsy Needle-GTA 20G, Quistello, Italy) was used for the puncture (Figure 1B). The diameter of obtained samples was 20G (0.812 mm). On the eightieth day after the biopsy in all of three groups of rats, under general anesthesia (procedure has been described previously) and sterile conditions funiculus has been ligated at the level of the external inguinal opening. The time frame of 80 days was chosen to determine the late immunohistochemical, histological and spermatogenetic consequences on the testes due to puncture biopsy. After that a scrotal skin incision was performed and all rats underwent bilateral orchiectomy and funiculectomy. Epididymal tail and the initial part of the vas deferens were removed and stored in vial with a solution for storage of sperm cells (Quinn’s Sperm Washing Medium, Sage In-vitro Fertilization, Inc., Trumbull, CT, USA) to obtain sperm for analysis. The remaining part of the epididymis and testis were fixed for 24 h in 4% buffered formaldehyde for histological and immunohistochemistry analyses. Upon completion of the experiment the animals were sacrificed.

### 2.3. Histological Analysis

**Figure 2:**
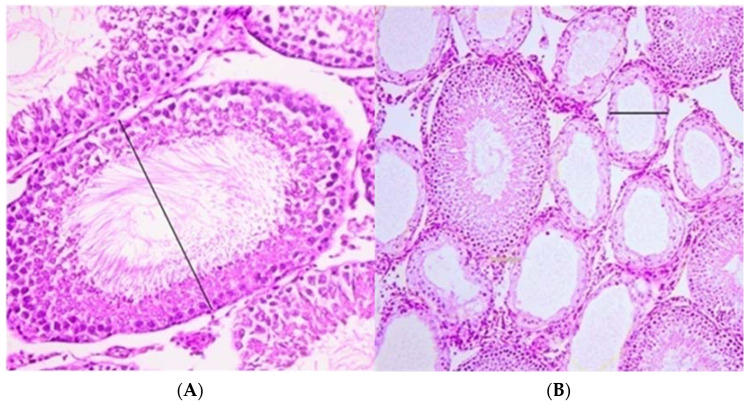
Histological measurement of tubules: (**A**)—normal tubules, HE, magnification ×40; (**B**)—atrophic tubules, HE, magnification ×20.

Testicular tissue, after fixation in 4% buffered formalin for 24 h, was processed using standard procedures of dehydration and waxing in histokinet Thermo Shandon (GMI Inc., Ramesey, MN 55303, USA). Afterwards, tissue was paraffin embedded and cut in 5-µm thick sections. The specimens for microscopy were mounted on slides, fixed in a thermostat for 15 min at 58–60 °C, deparaffinized by immersion for 30 min in xylol and rehydrated two times for 30 min in alcohol solutions of 100%, 96% and 70%, respectively. The sections were stained with standard hematoxylin-eosin method using an automated slide stainer (Sakura Tissue-Tek DRS 2000, Tokyo, Japan) and examined histologically. All tissue samples were analyzed by the same pathologist blinded to the assigned groups of prepubertal rats. Histological changes between the investigated groups were compared in terms of the number (mild—less than half of tubules are atrophic; moderate—more than half of tubules are atrophic; severe—all tubules are atrophic) and focality of atrophic seminiferous tubules (number of atrophic focuses or diffuse), the percentage of atrophy and the diameter of the tubules (Figure 2). Histological changes of seminiferous epithelium were analyzed according to Johnsen’s criteria [19,20].

### 2.4. Immunohistochemistry

**Figure 3:**
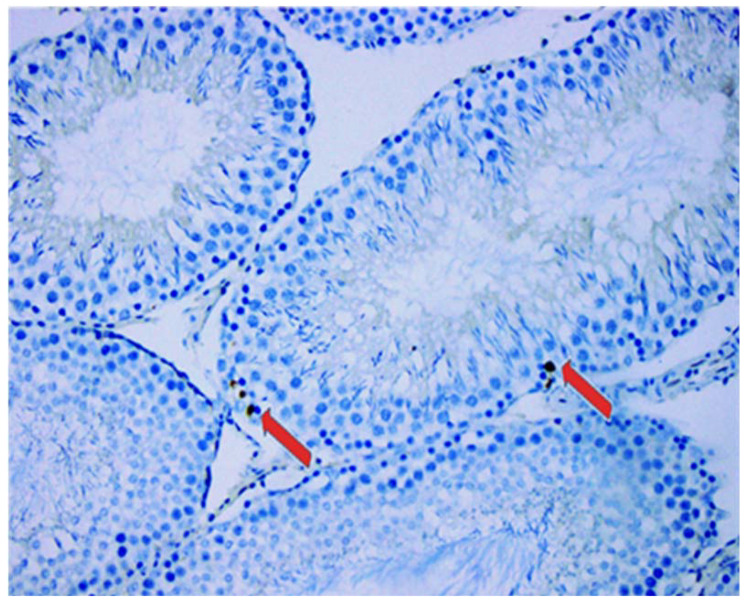
Immunohistochemical analysis of apoptotic activity. Positive immunostaining for cleaved caspase 3 in germ cells (red arrow), magnification ×40.

To measure apoptosis and apoptotic index the following immunohistochemical protocol was used. All paraffin blocks, with tissue from the central part of the testis, were cut at 5 µm, mounted on salinized slides and stained in an automated immunohistochemical slide stainer Ventana Benchmark. Incubation with primary rabbit antibody on rat cleaved caspase-3 (Asp175 cleaved caspase-3, Cell Signaling, Danvers, MA, USA) in concentration 1:500 was 60 min. A visualization system with chromogen diamino-benzidine tetrahydrochloridine were used. Brown intensive diffuse or granular nuclear expression was regarded as positive for caspase-3 antibody (Figure 3). The stained samples were analyzed with light microscope (BX 41, Olympus, Tokyo, Japan). Apoptotic cells were counted in germ cells as well as in somatic cells. The results were presented as the apoptotic index (AI), which represented the number of positively stained cells per hundred cells analyzed throughout 10 high power fields of view.

### 2.5. Sperm Extraction for Analysis of Sperm Count, Motility and Morphology

Epididymal tissue samples were incubated in a collagenase for 90 min at 37 °C in a CO_2_ incubator. After removal from the incubator the tissue was abundantly macerated with sterile needles and centrifuged at 500–800 *g* for 10 min. After removal of the supernatant with collagenase, the sample was resuspended in 0.5 mL of sperm-wash media and 10 µL of suspension was placed in a sperm counting chamber (Makler Counting Chamber, Sefi-Medical Instruments, Haifa 31070, Israel). Then, the number and motility of sperm and the percentage of abnormal forms of sperm were determined.

### 2.6. Statistical Analysis

Obtained data was analyzed using R software version 3.6.3 (R Core Team, Auckland, New Zealand) environment for statistical computing and graphics. Normality of investigated variables was checked using the Shapiro–Wilk test. If the analyzed variable, within all three groups, follows a normal distribution, the difference between the groups was tested by ANOVA or Welch one-way tests, depending on the homogeneity of variance. For homogeneity of variance Leven test was used. In the case of a statistically significant result, further analysis was performed by Tukey or Games–Howell post-hoc test. If the analyzed variable within one or more groups deviates from the normal distribution, the differences between the groups were tested by the Kruskal–Wallis test and in the case of a statistically significant result by the post-hoc Holm’s method. Comparisons between left and right testicles were performed using Student t test and Mann–Whitney U test. All the tests were two-sided and the significance level of 0.05 was used.

## 3. Results

In this study, statistical processing of several parameters for the left testicles, right testicles and, finally, both of them were performed. Statistically significant differences in following parameters for left testicle were found: volume (*p* < 0.001), sperm count (*p* < 0.05), apoptotic index (*p* < 0.01), percentage of atrophic tubules (*p* < 0.001) and Johnsen score (*p* < 0.001). The most important parameters for the left testicles are shown in Table 1.

**Figure 4:**
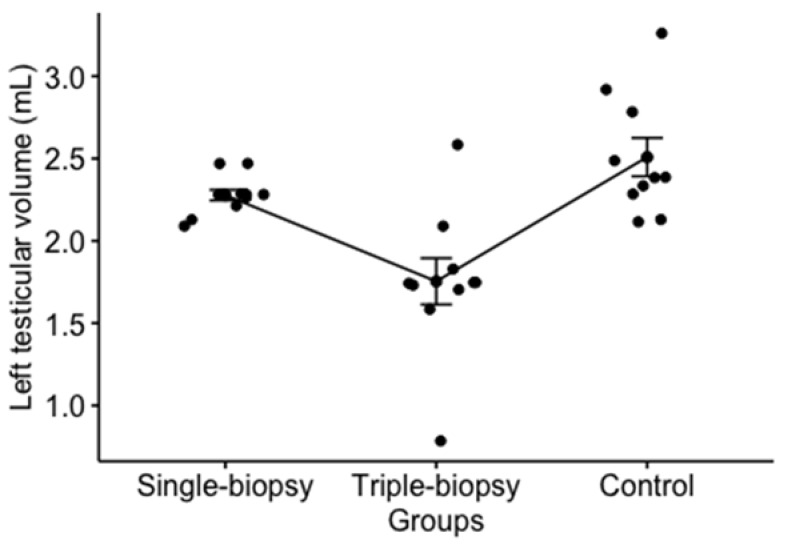
Difference in left testicular volume between the experimental groups (Kruskal-Wallis test; *p* < 0.001).

A significant difference in left testicle volume between experimental groups has been determined (*p* < 0.001). Post hoc analysis using the Holm’s method revealed a statistically significant difference between the single and triple-biopsy groups (*p* < 0.01); thus, the rats from the triple-biopsy group had a significantly smaller left testicular volume compared to the rats from single biopsy group. The same finding was noted between the triple-biopsy and control groups (*p* < 0.001) which shows that the volume of the left testicle of the rats from control group is significantly higher compared to the rats triple-biopsy group, while no significant difference was found between the rats from single-biopsy and control groups (*p* > 0.05) (Figure 4).

**Figure 5:**
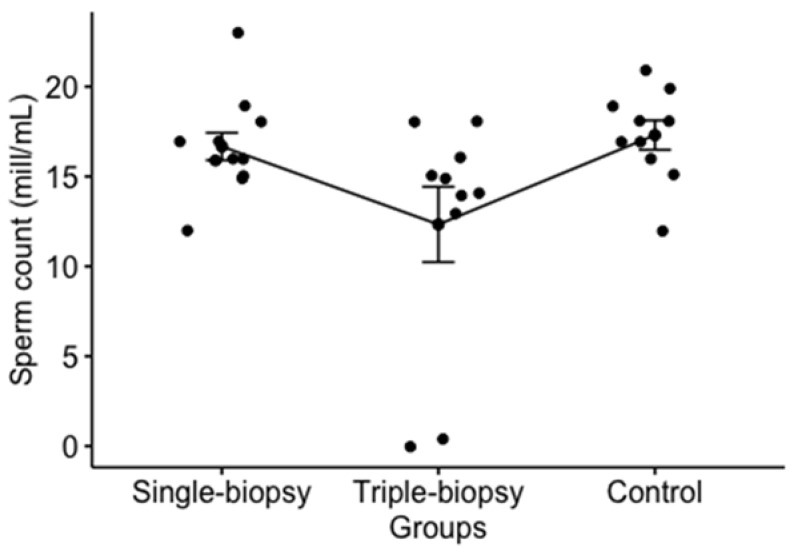
Left testis sperm count analysis (Kruskal-Wallis test; *p* < 0.05).

Sperm count analysis revealed a difference between the observed groups. Post hoc analysis showed that single-biopsy group did not have a statistically significantly different sperm count compared to triple-biopsy (*p* = 0.130) and control (*p* = 0.380) groups, while the sperm count between the triple-biopsy and control groups was of borderline statistical significance (*p* = 0.05) (Figure 5).

**Figure 6:**
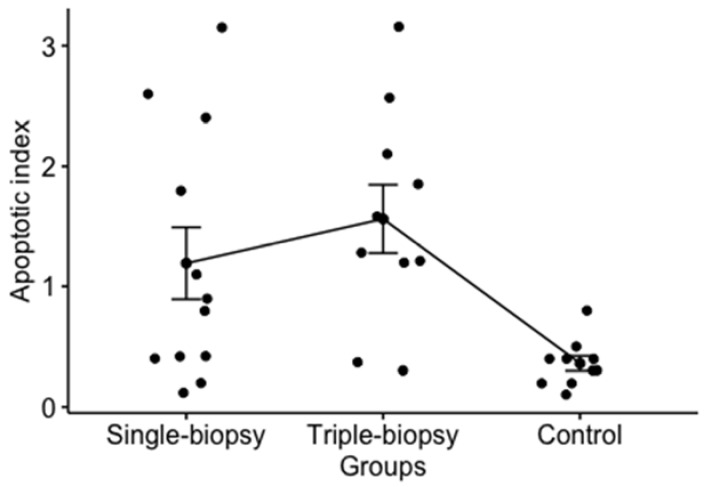
Apoptotic index of the left testicle between the experimental groups (Welch one-way test; *p* < 0.01).

Analysis of the apoptotic index showed a significant difference for left testis between the experimental groups (*p* < 0.01). Post hoc analysis using Games-Howell test determined statistically significant difference between the triple-biopsy and control groups (*p* < 0.01) and single-biopsy and control groups (*p* = 0.015). Using Games-Howell test higher apoptotic index in single and triple-biopsy groups compared to control group has been determined, while between the single and triple-biopsy groups no statistically significant difference was found (*p* = 0.650) (Figure 6).

**Figure 7:**
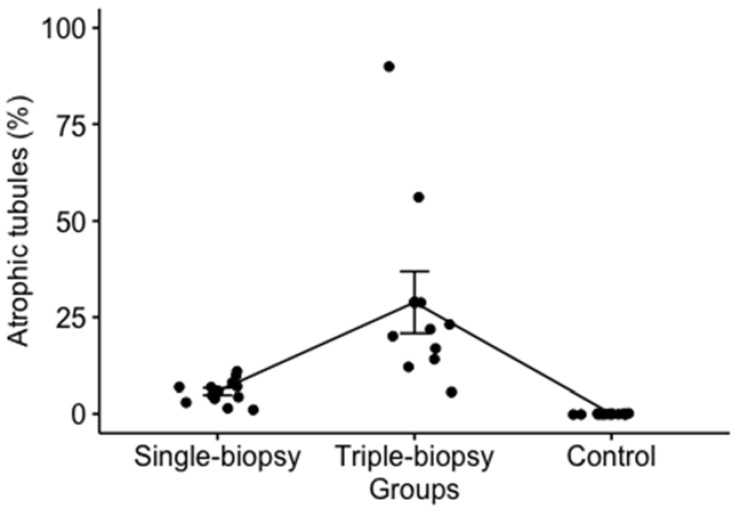
Percentage of atrophic tubules between the experimental groups (Kruskal-Wallis test; *p* < 0.001).

A statistically significant difference in the percentage of atrophic tubules between the experimental groups was found (*p* < 0.001), which was confirmed by post hoc analysis using the Holm’s method. The difference between the single and triple-biopsy groups (*p* < 0.001) has revealed statistically higher percentage of atrophic tubules in triple-biopsy group compared to the single-biopsy and control groups which had no atrophic tubules (*p* < 0.001). In addition, a statistically significant difference between the single and triple-biopsy groups was found (*p* < 0.001) (Figure 7).

**Figure 8:**
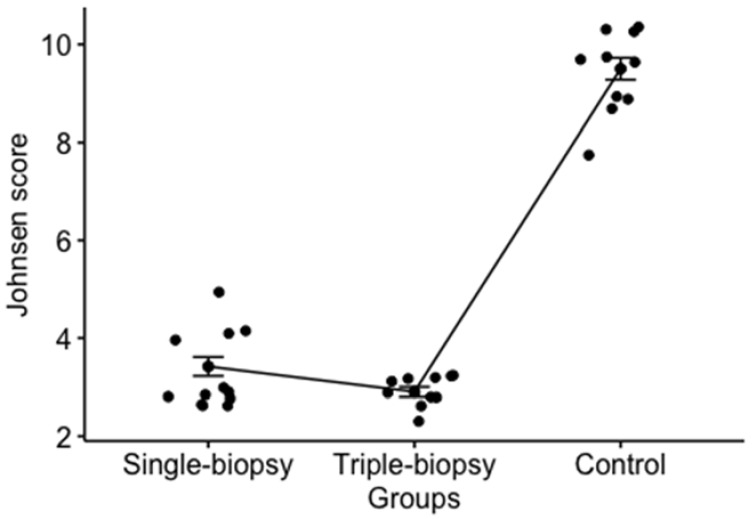
The Johnsen score value difference for the left testicle between the experimental groups (Kruskal-Wallis test; *p* < 0.001).

The value of Johnsen score for the left testicle between the experimental groups was shown as statistically significant parameter (*p* < 0.001). Post hoc analysis using Holm method showed statistically significant difference between all of the observed groups (single and triple-biopsy, *p* < 0.05; single-biopsy and control, *p* < 0.001; triple-biopsy and control, *p* < 0.001). Control group had the maximum score while single and triple-biopsy groups had differences in the Johnsen score (Figure 8). Despite the statistically significant difference between the single and triple-biopsy, this difference has no clinical value.

Statistical analysis did not reveal significant difference in the sperm motility (ANOVA; *p* > 0.05) as well as in the percentage of abnormal sperm of the left testis between the experimental groups (Kruskal-Wallis test; *p* > 0.05).

To perform appropriate control of the experiment the results of left testis, from which the biopsy was taken were compared with the right testis, which was left intact (Table 2).

**Figure 9:**
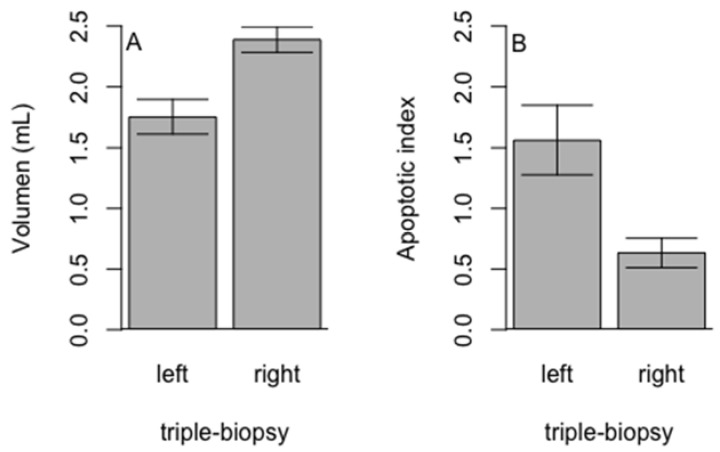
Comparison of the (**A**)—volume and (**B**)—apoptotic index of the left (punctured) and right (intact) testicles in triple biopsy group (Student *t*-test, *p* < 0.01).

In the triple-biopsy group volume of the right (intact) testicle was significantly higher compared to the volume of the left (punctured) testicle (*p* < 0.01) (Figure 9A). Significantly higher values of AI were found in left (punctured) testicles compared to the right (intact) testicles (*p* = 0.01) (Figure 9B).

**Figure 10:**
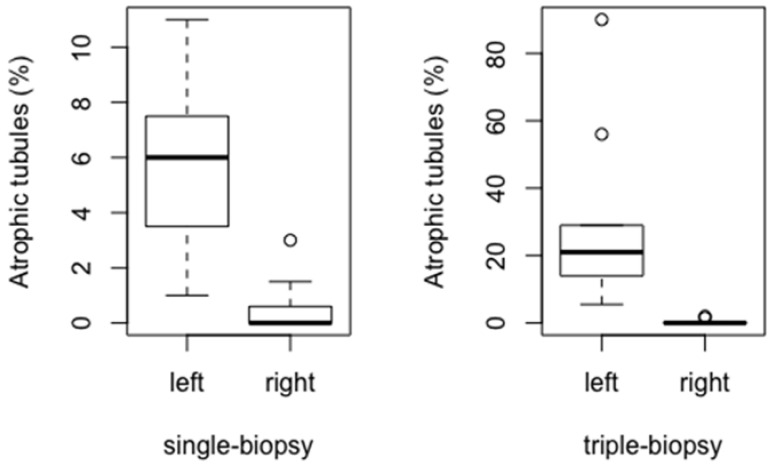
Comparison of atrophic tubules between the left and right testicles in single and triple-biopsy groups (Mann–Whitney *U* test, *p* < 0.001).

Higher percentage of atrophic tubules has been found in the group of left (biopted) testicles compared to the right (intact) testicles in single-biopsy (*p* < 0.001) and triple-biopsy (*p* < 0.001) groups (Figure 10).

**Figure 11:**
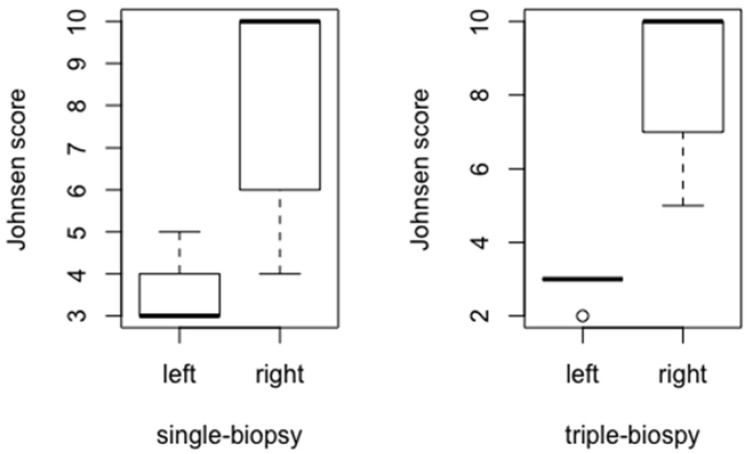
Comparison of Johnsen score between the left and right testicles in single and triple-biopsy groups (Mann-Whitney *U* test, *p* < 0.001).

The value of Johnsen score was significantly lower in the left (biopted) testicles compared to the right (intact) testicles in both single-biopsy (*p* < 0.001) and triple-biopsy (*p* < 0.001) groups (Figure 11).

## 4. Discussion

This study clearly proved that triple and especially the single testicular punch biopsy does not have gross effects on testicular function and sperm production in pubertal rats, suggesting that their fertility is most probably not impaired. For example, statistically significant difference in left testicular volume between biopsied left testicles and control group does not affect testicular function and sperm production in rats. The percentage of atrophic tubules in left testicles in triple punch group was significantly higher compared to single punch group. There were no atrophic tubules in the control group. Johnsen score values fort the left testicles and the total values of the Johnsen score for both testicles were statistically different. That means the control group had higher values compared to biopsy groups, indicating that there was preserved spermatogenesis of the control group and impaired spermatogenesis in the testicular tubules that were punctured. The apoptotic index was different in left as well as in both testicles, with significantly higher values in biopsy groups compared to the control group. Sperm analysis revealed no statistically significant difference in the left testicles. The reduced sperm count in triple punch biopsy group compared to control group reviled borderline significance, while in single punch biopsy compared to control group no significant difference was found which indicates less harmful single according to a triple needle biopsy. These findings are probably due to the small sample size, while on the larger sample the differences would be probably more expressed. No significant differences between the investigated groups in other parameters of sperm analysis, such as sperm mobility or morphological abnormality, which could indicate a possible decrease in fertility, have been found. Analysis of the observed parameters showed that histological and immunohistochemical changes of punctured testicles do not have a significant effect on the spermiogram and in particular on the overall fertility.

Testicular biopsy is an important diagnostic tool in evaluation of male infertility, especially in terms of the assessment of fertility in men with azoospermia and oligospermia and determination of the ductal obstruction compared to primary testicular disorders [12]. In addition to the evaluation of male infertility, this method is also very useful in sampling for ICSI, screening of testicular cancer or monitoring of chemotherapy responses in children with acute lymphocytic leukemia [7,13]. The applicability of this method causes significant controversy in pediatric population, but testicular biopsy is very useful for the assessment of testicular damage in boys with cryptorchidism, varicocele, testicular torsion or testicular injury [21,22,23,24,25]. Several published studies agreed that findings obtained from testicular punch biopsy could have a good prognostic value for treatment of varicocele [12,18,19,20]. The controversy of this method is the fact that biopsy may cause a disorder in testicular maturation and development, especially if the biopsy is performed at earlier age [3,18]. For a long time, the standard method for obtaining testicular tissue samples was an open biopsy, but since the nineties of the last century, in human diagnostics and in various animal models, punch biopsy began to be used more frequently and progressively replaced open biopsy. The validity of punch biopsy in regards to quality of obtained samples was compared with open biopsy in several studies. Kessaris and Morey in their studies have shown a pathohistological correlation of 95–98% with the traditional open biopsy [7,8].

To obtain an appropriate sample for histological analysis, it is necessary to use the appropriate needle with the appropriate diameter. Rosenlund et al. in their study showed that a 19G needle diameter used for the biopsy was as good as an open biopsy [13]. According to Nakane et al., the amount of testicular tissue taken from rats in an open biopsy should not exceed 3% of the total testis weight in order to avoid harmful consequences for the testis. This amount of testicular tissue is enough for the analysis [3]. In humans, the volume of the sample taken at biopsy is generally less than 1% of the total testicular weight, which significantly reduces the possibility of testicular damage [3]. In adult rat model Cosentino et al. proved that the removal of a relatively large amount of testicular tissue in unilateral biopsy transiently affects reproductive ability without visible effects on the opposite testis [26,27,28]. Many factors may affect the consequences caused by open or punch biopsy. Except the type of biopsy there are several factors that may affect the final outcome such as the size of needle, age, localization, direction and number of biopsies [27,28,29,30].

According to the previous knowledge based on animal experiments there is no a firm stand about the consequences of testicular biopsy on spermiogenesis. Still, there is not enough evidence with the standardized model of experiment performance and standardized measurements. From the studied literature, it can be concluded that biopsies performed on larger animals (e.g., dogs, cats) compared to rats’ causes minor consequences.

For definitive recommendations in regards to investigated procedure, as a standard diagnostic method to be used in human medicine, further studies on larger sample size should be performed. An observation that minimal immunohistochemical changes were present on the non-biopsied right testes, without a clear explanation, may be the subject of new studies.

## 5. Conclusions

Nowadays, a punch biopsy is a simple, reliable and inexpensive method for different types of tissue sampling. Equally, it is the method of choice for obtaining testicular tissue samples for pathohistological analysis and sperm for ICSI. Our results clearly showed that a single biopsy has no gross effects on testicular function and sperm production. Triple biopsy showed by the same parameters that histological and immunohistochemical consequences were more significant compared to single but without a significant effect on overall testicular function and sperm production. Sperm analysis showed that single and triple biopsies did not have a significant effect on sperm count, motility and morphology. Single and triple punch biopsies of one testicle did not significantly affect the overall fertility potential of pubertal rats.

## Figures and Tables

**Table 1:** Examined variables for left testicles.

Groups Variables		Single and Triple-Biopsy			Single-Biopsy and Control			Triple-Biopsy and Control		
	Statistical Analysis	Median (IQR) or Mean ± SD		Post-Hoc Test	Median (IQR) or Mean ± SD		Post-Hoc Test	Median (IQR) or Mean ± SD		Post-Hoc Test
Volume (mL)	H = 15.6; *p* < 0.001 ^a^	2.28 (2.25–2.29)	1.7 (1.71–1.81)	z = 3.6; *p* < 0.01 ^d^	2.28 (2.25–2.29)	2.39 (2.29–2.71)	z = 2.1 *p* = 0.052 ^d^	1.7 (1.71–1.81)	2.39 (2.29–2.71)	z = 4.8 *p* < 0.001 ^d^
Sperm count (mill/mL)	H = 6.5; *p* < 0.05 ^a^	16 (15.75–17.25)	14.5 (13.25–15.75)	z = 1.9; *p* = 0.13 ^d^	16 (15.75–17.25)	17.5 (16.25–18.75)	z = 2.6 *p* = 0.380 ^d^	14.5 (13.25–15.75)	17.5 (16.25–18.75)	z = 2.6 *p* = 0.05 ^d^
Sperm motility (%)	(F = 3.1; *p* > 0.05) * F = 1.5; *p* > 0.05 ^b^	32.7 ± 15.8	24.6 ± 18.4	-	32.7 ± 15.8	35.4 ± 7.6	-	24.6 ± 18.4	35.4 ± 7.6	-
Abnormal sperm (%)	H = 4.3; df = 2; *p* > 0.05 ^a^	27 (25.5–29)	30 (28–33.5)	-	27 (25.5–29)	31 (28.5–35.5)	-	30 (28–33.5)	31 (28.5–35.5)	-
Apoptotic index	(F = 4.9; *p* < 0.05) * F = 11.2; *p* < 0.01 ^c^	1.19 ± 1.04	1.56 ± 0.9	t = 0.89; *p* = 0.65 ^e^	1.19 ± 1.04	0.36 ± 0.19	t = 2.72 *p* = 0.015 ^e^	1.56 ± 0.9	0.36 ± 0.19	t = 4.1 *p* < 0.01 ^e^
Atrophic tubules (%)	H = 26.9; *p* < 0.001 ^a^	6 (3.75–7.25)	21 (14.75–27.5)	z = 5.5; *p* < 0.001 ^d^	6 (3.75–7.25)	0	z = 9.8; df = 11; *p* < 0.001 ^d^	21 (14.75–27.5)	0	z = 15.6 *p* < 0.001 ^d^
Johnsen score	H = 25.2; *p* < 0.001 ^a^	3 (3–4)	3(3–3)	z = 2.54; *p* < 0.05 ^d^	3 (3–4)	10 (8–10)	z = 8.2 *p* < 0.001 ^d^	3(3–3)	10 (8–10)	z = 14.9 *p* < 0.001 ^d^

* Levene test; ^a^—Kruskal-Wallis test; ^b^—ANOVA; ^c^—Welch’s ANOVA; ^d^—Holm’s method; ^e^—Games-Howell test; IQR—Interquartile range; SD—Standard deviation.

**Table 2:** Comparison between left (punctured) and right (intact) testicles in single and triple-biopsy groups.

Group Variables	Single-Biopsy			Triple-Biopsy		
	Median (IQR) or Mean ± SD		*p*	Median (IQR) or Mean ± SD		*p*
	Left Testis	Right Testis		Left Testis	Right Testis	
Volume (mL)	2.28 (2.25–2.29)	2.28 (2.28–2.52)	0.36	1.74 ± 0.44	2.38 ± 0.33	<0.01
Sperm count (mill/mL)	16.7 ± 2.64	16.5 ± 2.39	0.87	14.5 (13.25–15.75)	15.25 (13.25–17.5)	0.47
Sperm motility (%)	32.7 ± 15.8	34.6 ± 18.1	0.79	24.6 ± 18.4	29.1 ± 17.6	0.58
Abnormal sperm (%)	28 ± 4	27.8 ± 5.1	0.93	30 (28–33.5)	31 (26.5–34)	0.85
Apoptotic index	1.19 ± 1.04	0.59 ± 0.29	0.08	1.56 ± 0.9	0.63 ± 0.38	0.01
Atrophic tubules (%)	6 (3.75–7.25)	0 (0–0.4)	<0.001	21 (14.75–27.5)	0 (0–0.23)	<0.001
Johnsen score	3 (3–4)	10(6.5–10)	<0.001	3 (3–3)	10 (7.25–10)	<0.001

IQR—Interquartile range; SD—Standard deviation.

## Data Availability

The data presented in this study is available upon request of the respective author. Due to the protection of personal data, the data is not publicly available.

## References

[1] Kang C., Punjani N., Schlegel P.N. (2021). Reproductive chances of men with azoospermia due to spermatogenic dysfunction. J. Clin. Med..

[2] Pogorelić Z., Sopta M., Jukić M., Nevešćanin A., Jurić I., Furlan D. (2017). Laparoscopic varicocelectomy using polymeric ligating clips and its effect on semen parameters in pediatric population with symptomatic varicocele: A 5-year single surgeon experience. J. Laparoendosc. Adv. Surg. Tech. A.

[3] Nakane A., Kojima Y., Hayashi Y., Kurokawa S., Mizuno K., Kohri K. (2005). Effect of testicular biopsy in childhood on spermatogenesis, fertility, and paternity in adulthood—A mouse model study. Urology.

[4] Uygur M.C., Arik A.I., Erol D., Ozer E., Ustün H. (1999). Quantitative evaluation of biopty gun testis needle biopsy. Correlation between biopsy score of varicocele-bearing testis and sperm count. J. Reprod. Med..

[5] Faure A., Bouty A., O’Brien M., Thorup J., Hutson J., Heloury Y. (2016). Testicular biopsy in prepubertal boys: A worthwhile minor surgical procedure?. Nat. Rev. Urol..

[6] Elzanaty S. (2014). Varicocele repair in non-obstructive azoospermic men: Diagnostic value of testicular biopsy—A meta-analysis. Scand. J. Urol..

[7] Kessaris D.N., Wasserman P., Mellinger B.C. (1995). Histopathological and cytopathological correlations of percutaneous testis biopsy and open testis biopsy in infertile men. J. Urol..

[8] Morey A.F., Deshon G.E., Rozanski T.A., Dresner M.L. (1993). Technique of biopty gun testis needle biopsy. Urology.

[9] Cito G., Coccia M.E., Sessa F., Cocci A., Verrienti P., Picone R., Fucci R., Criscuoli L., Serni S., Carini M. (2019). Testicular Fine-Needle Aspiration for Sperm Retrieval in Azoospermia: A Small Step toward the Technical Standardization. World J. Men’s Health.

[10] Carpi A., Fabris F.G., Todeschini G., Nardini V. (2006). Large-needle percutaneous aspiration biopsy of the testicle in men with nonobstructive azoospermia. Fertil. Steril..

[11] Rajfer J., Binder S. (1989). Use of biopty gun for transcutaneous testicular biopsies. J. Urol..

[12] Lee A.P., Roth M.Y., Nya-Ngatchou J.J., Lin K., Walsh T.J., Page S.T., Matsumoto A.M., Bremner W.J., Amory J.K., Anawalt B.D. (2016). Testicular fine-needle aspiration for the assessment of intratesticular hormone concentrations. Asian J. Androl..

[13] Cohen M.S., Frye S., Warner R.S., Leiter E. (1984). Testicular needle biopsy in diagnosis of infertility. Urology.

[14] Ramanathan S., Dogra V. (2018). Current status of percutaneous testicular biopsy for focal lesions. Abdom. Radiol..

[15] Schlegel P.N., Su L.M. (1997). Physiological consequences of testicular sperm extraction. Hum. Reprod..

[16] Altay B., Hekimgil M., Kefi A., Girgin C., Cikili N. (2000). A comparison of the histopathological findings after open and percutaneous needle testicular biopsy in adult male rats. BJU Int..

[17] Shufaro Y., Prus D., Laufer N., Simon A. (2002). Impact of repeated testicular fine needle aspirations (TEFNA) and testicular sperm extraction (TESE) on the microscopic morphology of the testis: An animal model. Hum. Reprod..

[18] Stampfli M., Hadziselimovic F. (1991). The effect of needle biopsy procedure on prepubertal rat testis. Eur. J. Pediatr Surg..

[19] Johnsen S.G. (1970). Testicular biopsy score count-a method for registration of spermatogenesis in human testes: Normal values and results in 335 hypogonadal males. Hormones.

[20] Dohle G.R., Elzanaty S., van Casteren N.J. (2012). Testicular biopsy: Clinical practice and interpretation. Asian J. Androl..

[21] Todorić D., Durdov M.G., Tandara M., Čapkun V., Jurić I., Biočić M., Meštrović J., Pogorelić Z. (2014). Influence of open testicular biopsy in prepubertal rats on rats' adulthood fertility with correlation to serum levels of inhibin B and follicle stimulating hormone. J. Pediatr. Urol..

[22] Pogorelić Z., Jurić I., Biočić M., Furlan D., Budimir D., Todorić J., Milunović K.P. (2011). Management of testicular rupture after blunt trauma in children. Pediatr. Surg. Int..

[23] Jukic M., Todoric M., Todoric J., Susnjar T., Pogorelic Z. (2019). Laparoscopic versus open high ligation for adolescent varicocele: A 6-year single center study. Indian Pediatr..

[24] Pogorelic Z., Neumann C., Jukic M. (2019). An unusual presentation of testicular torsion in children: A single—Centre retrospective study. Can. J. Urol..

[25] Pogorelić Z., Mrklić I., Jurić I., Biočić M., Furlan D. (2013). Testicular torsion in the inguinal canal in children. J. Pediatr. Urol..

[26] Cosentino M.J., Sheinfeld J., Erturk E., Cockett A.T. (1986). The effect of graded unilateral testicular biopsy on the reproductive capacity of male rats. J. Urol..

[27] Gul S.B., Polat A.V., Bekci T., Selcuk M.B. (2016). Accuracy of percutaneous CT-guided spine biopsy and determinants of biopsy success. J. Belg. Soc. Radiol..

[28] Rohländer M., Otzen H., Rode K., Jung K., Schmicke M., Harborth T., Langeheine M., Brehm R., Bajcsy Á.C. (2020). Histological comparison of testicular needle biopsy and en bloc samples in abattoir calves. Animals.

[29] Odabaş O., Uğraş S., Yilmaz Y., Aydin S., Atilla M.K. (1997). Testicular needle biopsy: Is it a safe and adequate method?. Int. Urol. Nephrol..

[30] Murshidi M.M., Choy J.T., Eisenberg M.L. (2020). Male infertility and somatic health. Urol. Clin. N. Am..

